# Unplanned Readmission within 28 Days of Hospital Discharge in a Longitudinal Population-Based Cohort of Older Australian Women

**DOI:** 10.3390/ijerph17093136

**Published:** 2020-04-30

**Authors:** Dinberu S. Shebeshi, Xenia Dolja-Gore, Julie Byles

**Affiliations:** 1 Research Centre for Generational Health and Ageing (RCGHA), Faculty of Health and Medicine, The University of Newcastle, Newcastle 2308, Australia; xenia.doljagore@newcastle.edu.au (X.D.-G.); julie.byles@newcastle.edu.au (J.B.); 2 Centre for Clinical Epidemiology and Biostatistics, University of Newcastle, Newcastle 2308, Australia; 3 Research Assets Division, SAX Institute, Level 3, 30C Wentworth Street, Glebe, NSW 2037, Australia

**Keywords:** Australia, Cox regression, older people, unplanned readmission, women

## Abstract

This study aimed to estimate the incidence of 28-day unplanned readmission among older women, and associated factors. Data were used from the 1921–1926 birth cohort of the Australian Longitudinal Study on Women’s Health. Linkage of self-reported survey data with the Admitted Patient Data Collection allowed the identification of hospital admissions for each woman and the corresponding baseline characteristics. The Cox proportional-hazards model was used to identify factors associated with time to unplanned readmission, using SAS software V 9.4. (SAS Institute, Cary, NC, USA). Of 2056 women with index unplanned admission, 363 (17.5%) were readmitted within 28 days of discharge, and of these 229 (11.14%) had unplanned readmission. Among women with unplanned readmission, 24% were for the same condition as for the index hospitalisation. Cardiovascular diseases were the main diagnoses for the index admission and readmission. Unplanned readmission risk was higher if not partnered (hazard ratio (HR) = 1.43, 95% confidence interval (CI): 1.05–1.95), of non-English speaking background (HR = 1.62%, 95% CI: 1.07–2.47), more than three days length of stay on index admission (HR = 1.41%, 95% CI: 1.04–1.90) and one or two of the assessed chronic diseases (HR = 1.68, 95% CI: 1.19–2.36). At least one in ten women had unplanned readmission at some time between ages 75–95 years. Women who are not partnered, not of English-speaking background, with longer hospital stay and those with multi-morbidity, may need further efforts during their stay and on discharge to mitigate unplanned readmission.

## 1. Introduction

In Australia, older people account for a high proportion of hospital users. In 2016–2017, 42% of separations and 48% of patient days were for people aged 65 and over [1]. Older patients also are most likely to have rapid return or readmission within a few days of hospital discharge [2]. Unplanned readmissions may be associated with hospitalisation-acquired complications, higher use of hospital and community healthcare resources, and faster functional decline [3]. Furthermore, unplanned readmission is an additional health burden and costly for older populations, and therefore a challenge for healthy ageing. A significant proportion of readmission would be avoidable and potentially preventable, through identifying patients at greatest risk of readmission, and providing focussed attention on their needs during their admission and on discharge [4].

In Australia, rates of unplanned readmission within 28 days of hospital discharge are deemed to be an indicator of healthcare quality and safety [1,5]. For instance, in the state of New South Wales (NSW), the 2016–2017 unplanned readmission rate was 35.8 per 1000 separations following tonsillectomy and adenoidectomy, and 27.9 per 1000 separations following hysterectomy [1]. Furthermore, a study from a major health service in Victoria showed that the unplanned readmission rate within 28 days of discharge from acute care was 7.4%, and significantly higher among people aged 65 years and over [5]. Therefore, identifying factors contributing to unplanned readmission is a key public health priority.

Our review of published studies shows wide variations in the rate and contributing factors of readmission. Although the reason for these variations is not often reported, these inconsistencies appear to be attributable to a lack of uniformity in defining a time-period to identify readmission. For instance, in Victoria, Australia, a study showed that 11.1% of unplanned readmissions within one day of discharge were associated with discharge against advice, or with repeated hospital admission in the months preceding the index admission [6]. Another study found that 60 days unplanned readmission was significantly associated with post-discharge environmental and socioeconomic factors such as living alone, having an unmet functional need, lacking self-management skills and poor education [7]. Overall, the definition of the readmission varied from one day to 60 days after the discharge date of the index hospital admission [6,7]. The unplanned readmission rates in older Australians (based on 28-day readmission) vary from 7.4% to 24.9% [5,6,8,9]. Significant predictors of readmission among older people were the length of stay in index admission, chronic disease burden, not partnered, lower education, female sex, lack of self-management, living rural, lack of social support, and incorrect or discontinued use of medicine.

The main limitation of the previous studies was the operational definition of the unplanned readmission and restriction to a specific diagnostic group. There is also a lack of distinction between unplanned and planned readmission. Indeed, the reasons for unplanned readmission may be different from the planned readmission [10]. To mention a few, studies have assessed risk factors of older people’s unplanned readmission following the diagnosis of cancer [11], hip arthroplasty [12], congestive heart failure [13], admission to medical geriatric rehabilitation unit [14], lung resection [15] and excisional breast surgery [16]. However, older people are an increasingly important population in hospitals, and are likely to have frequented hospital readmissions for various issues including multiple morbidities [17]. Following experience of unplanned hospital admission, older people are at high risk of functional decline, adverse events, increased needs for care, additional care service use, and quick return to hospital [18]. Therefore, it is worthwhile to identify factors contributing to unplanned readmission in later life following discharge from unplanned hospitalisation episodes, regardless of diagnoses.

There is little evidence from prospective studies that assess the rate and predictors of unplanned readmission in later life. Many studies have a cross-sectional rather than a longitudinal design [19,20]. Several are also restricted to a specific healthcare institution [6,14,21] or condition [11,12,16]. This gap presents a need for findings from reliable study data for the general population of older people. To address the gap, this study linked self-reported surveys of participants from the 1921–1926 birth cohort of the Australian Longitudinal Study on Women’s Health (ALSWH) [22] with Admitted Patient Data Collection (APDC) to identify unplanned readmission incidence. In general, this study aimed to assess the rate of unplanned readmission within 28 days of discharge from unplanned hospitalisation, and associated risk factors within a large cohort of older women.

## 2. Materials and Methods

### 2.1. Data

The data were taken from the Australian Longitudinal Study on Women’s Health’s (ALSWH) 1921–1926 cohort [22]. The ALSWH is an ongoing prospective, national population-based study designed to assess factors that influence women’s physical and mental health, as well as psychosocial aspects of health (i.e., socio-demographic and lifestyle factors) and their health service use. The study commenced in 1996, when women in three age groups (born 1973–1978, 1946–1951 and 1921–1926) were randomly selected from the universal national health insurance scheme known as Medicare. Medicare contains the name and addresses information of Australian citizens as well as permanent residents. Women from rural and remote areas were sampled twice as much as women from urban areas, to provide sufficient representation of women outside major cities and to ensure appropriate statistical power for comparison in this group [23].

In 1996, 12,432 women from the 1921 to 1926 cohort completed the baseline survey (when aged 70–75 years). The cohort has been surveyed every three years since 1996 (1999, 2002, 2005, 2008, 2011), and surviving women have been surveyed every six months since 2011. ALSWH participants in the 1921–1926 cohort are representative of the population of women in their age group nationally, with slight over-sampling of married, Australian born and tertiary-educated women [24]. This study included women who were New South Wales (NSW) residents from 2001 to 2011 and consented to link their survey with hospital data (*n* = 3739).

### 2.2. Hospital Data

Australia’s hospital care is mainly financed by general taxation at national and state levels and managed by the states. The NSW Admitted Patient Data Collection (APDC) includes admission and separation dates, the urgency of the admission, and principal diagnosis for all admissions to public and private hospitals in NSW. For this study, APDC from 2001–2016 was used to identify admissions among the participants. The cause of admission has been provided according to the International Classification of Diseases, Tenth Revision, Australia Modification (ICD-10-AM) principal diagnosis codes [25]. Information about the urgency status is also available for each admission, and classified as emergency/unplanned, non-emergency/planned, urgency not assigned, maternity/newborn and regular same day planned admissions. Furthermore, the mode of separation was recoded as discharged by hospital, discharge on leave, transferred to palliative care, discharge at own risk, transferred to the nursing home, transferred to the psychiatric hospital, transferred to another hospital, and died. This study used the first unplanned admission for all women with unplanned overnight admissions as an index admission. The outcome is unplanned readmission within 28 days of alive discharge from the overnight stay in unplanned hospitalisation.

Of 3739 women residents in NSW, 426 had day admissions only, 2816 had at least one unplanned overnight hospitalisation in 2001–2016, and 2056 had an overnight unplanned admission where they were discharged alive by the hospital. Reasons for index admissions were collated using the primary diagnosis code using ICD-10-AM [25]. The ICD-10-AM codes were recoded into broader disease categories, based on ICD-10-AM groupings. For instance, “I30–I52” refers to ‘other forms of heart disease’, “I20–I25” refers to ‘ischaemic heart disease’ and “R00–R09” refers to ‘symptoms and signs involving the circulatory and respiratory systems’. Using these categories, we presented mostly reported principal diagnoses for index and unplanned readmission.

### 2.3. Covariates

Patients’ ages were presented by year. Covariates were extracted from the latest survey before the date of index admission and filling missing data from the previous surveys where necessary. Marital status was categorised as partnered (married and de facto relationship) and not partnered (separated, divorced and widowed). Area of residence, derived from the women’s home address, was categorised using standard Accessibility and Remoteness Index of Australia (ARIA+) classification as metropolitan, inner regional and outer regional/remote/very remote [26]. Private insurance was dichotomised: yes and no. English speaking status was categorised as a native English speaker and not principally English speaker (or non-English speaking background). Education level categorised as three categories: no formal education, school certificate and higher/above school certificate.

Body mass index (BMI) was computed as weight (kg) divided by height (m^2^), and categorised as: underweight (<18.5 kg/m^2^), normal weight (18.5–24.9 kg/m^2^), overweight (25–29.9 kg/m^2^) or obese (≥30 kg/m^2^) according to the World Health Organization classification [27]. Women’s smoking status was categorised as a non-smoker/never smoked, ex-smoker, and current smoker. Length of hospital stay (LOS) during the index admission was categorised as less than or equal to three days and more than three days. The number of general practitioner (GP) or family doctor visits within 12 months before the index admission was categorised as less or equal to four visits and greater than four visits [28]. Self-reported pre-existing chronic disease or conditions were categorised as ‘no’, 1–2, and more than two of the six chronic diseases (hypertension, diabetes, heart disease, breast cancer, stroke and asthma).

Covariates were included according to Andersen’s behavioural model structure for understanding factors that lead to the use of health services [29]. These include *predisposing* (i.e., age and marital status), *enabling* (i.e., area, education, insurance) and *need* (smoking, length of hospital stay during the index admission, number of general practitioner (GP or family doctor) visits, BMI, perceived general health and chronic condition burdens) factors.

### 2.4. Statistical Analysis

Survival time was measured in days, and patients were censored at 28 days from the discharge date. Descriptive analysis was performed to compare patients’ characteristics with readmission status using Pearson’s chi-square for categorical variables and Kruskal-Wallis test for continuous variables. Kaplan-Meier survival curves were used to estimate the risk of unplanned readmission within 28 days after discharge for categorical variables. The log-rank test was used to compare the survival experience of patients with different characteristics. Semi-Parametric Cox regression was used to estimate the hazard ratio of the covariates. Under the semi-parametric survival model, the risk of admission at time t for a particular individual with a set of *p* covariates (*x_1_*, *x_2_*, …, *x_p_*) is given as follows [30].

$$h\left(t|X\right)=h_{0}\left(t\right)\exp(\beta_{1}x_{1}+\beta_{2}x_{2}+\ldots +\beta_{p}x_{p})=h_{0}\left(t\right)\exp(\beta^{\prime }X)$$

where, $\beta_{i}$ is the estimated parameter for the $i^{th}$ covariate, $h_{0}\left(t\right)$ is the baseline hazard function, and *X* is the vector of covariates. Statistical models are used to link the study outcome with one or more predictor variables and examine the association strength between them [31]. In this study, we performed univariate analysis for all sets of predictor variables. Additionally, we included variables which were significant at the 0.25 significance level into multivariate analysis [31]. To evaluate predictor variables at the univariate level, we have assessed overall (global) *p*-value. Patients’ ages at index admission remained in the model as they are clinically important to observe hospital admission trajectories by age. Furthermore, models have been adjusted by the residence area of the patients due to the sampling nature of the survey [23]. Additional analysis has been carried out by including all readmission incidences within 28 days of discharge (i.e., unplanned readmission, planned readmission and urgency not assigned flagged readmissions). The sensitivity analysis was performed by including the length of time between the women’s most recent survey (which will vary according to the women) and the date of index admission in the multivariate analysis.

The basic assumption of the proportional hazard model is that the hazard ratios are constant over time. This means the risk of failure is the same no matter how long subjects or individuals have been followed. Therefore, the proportional hazard model assumption was checked by creating an interaction between variables and the logarithm of survival time (time-dependent covariate) and checking the significance of the hazard ratio. Model fittings were undertaken using the statistical software SAS v 9.4. (SAS Institute, Cary, NC, USA).

### 2.5. Ethical Clearance

The ALSWH project has ongoing ethical clearance from both the University of Newcastle (H-076-0795 and H-2012-0256) and the University of Queensland’s (2004000224 and 2012000950) Human Research Ethics Committees. Linkage of ALSWH survey data to the NSW APDC ethical clearance was obtained from the NSW Population and Health Services Research Ethics Committees. Ethics approval for the linkage of ALSWH self-reported survey data to the National Death Index (NDI) was obtained from the AIHW Ethics Committee.

## 3. Results

**Figure 1:**
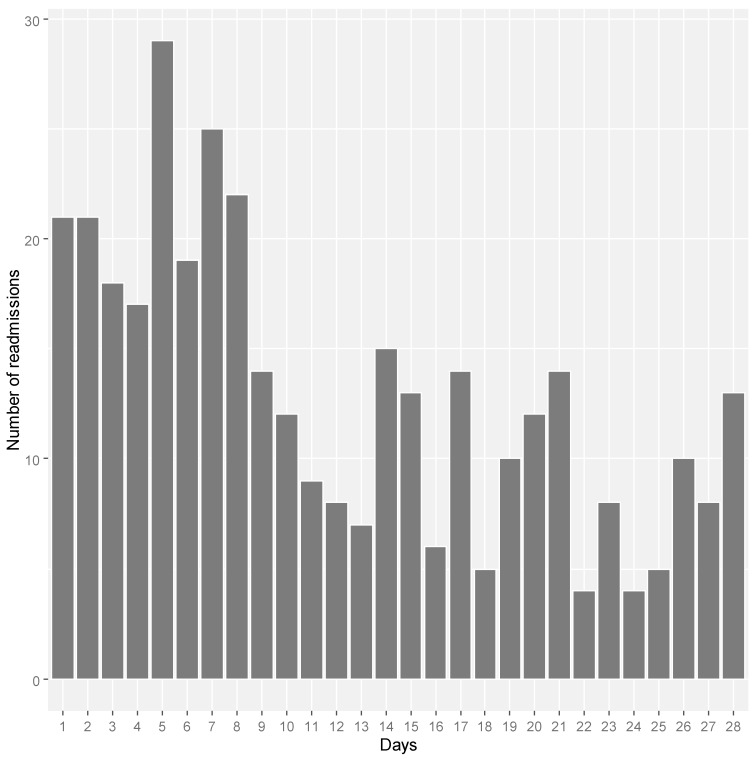
The number of readmissions within 28 days of discharge date by readmission day, in women aged 75 years and above (*n* = 363) from 1921 to 1926 birth cohort of ALSWH (Australian Longitudinal Study on Women’s Health).

Overall, 2056 of eligible women had at least one unplanned overnight hospital admission where they were discharged alive from the hospital during the period 2001–2016. During the index admission, the women had an average age of 82.1 years with a standard deviation of 3.96 years (Table 1). The median length of hospital stay during index admission was nearly 4 days (interquartile range (IQR): 2–8 years). Of the 2056 women, 8 women died and 363 (17.7%) women had readmission 28 days post-discharge. Of readmitted patients, 229 (11.14%) and 134 (6.52%) patients had unplanned and planned readmission respectively. Many readmissions (41.3%) occurred within 7 days of discharge (Figure 1).

**Figure 2:**
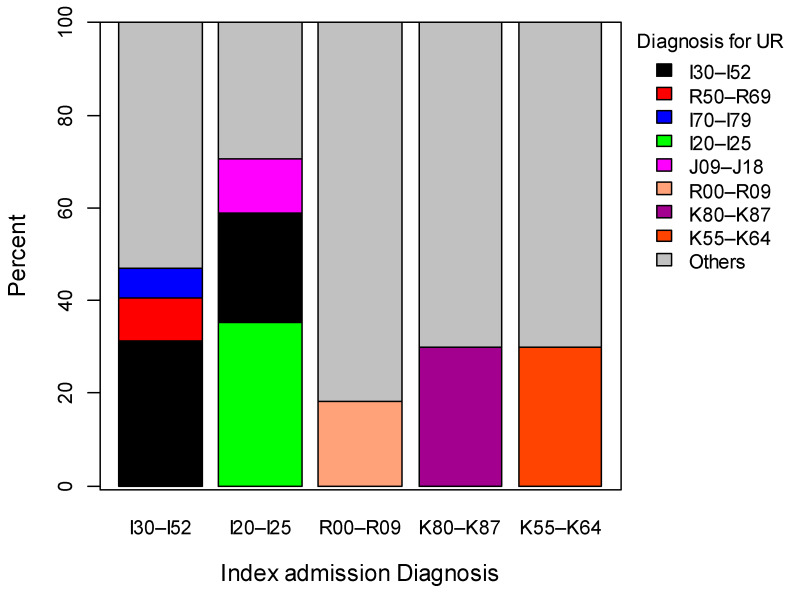
Diagnosis for index admission and unplanned readmission in women aged 75 years and over from 1921 to 1926 birth cohort of ALSWH. UR—unplanned readmission; I30–I52 are other forms of heart disease; R50–R69 are General symptoms and signs; I70–I79 are Arterial embolism and thrombosis; I20–I25 are Ischaemic heart disease; J09–J18 are Influenza and pneumonia; R00–R09 are Symptoms and signs involving the circulatory and respiratory systems; K80–K87 are Disorder of gallbladder biliary tract and pancreas; K55–K64 are Other diseases of intestines.

There were significant differences between women with no readmission, planned readmission and unplanned readmission for marital status, language, smoking, and the number of chronic conditions (Table 1). The top three diagnoses of unplanned readmission, among the top five diagnoses of index admission, were presented (Table 2, Figure 2). Nearly one in five women were readmitted with the same condition as for the index admission. The main reasons for planned readmission were health service for specific procedures and healthcare, other diseases of intestine and other forms of heart diseases.

### Predictors of Unplanned Readmission within 28 Days of Discharge

After adjusting potential variables, patient’s marital status, English speaking background, length of hospital stays during index admission and living with chronic disease were significant variables and remained in the model.

For instance, risk of unplanned readmission is higher by 43% among not partnered patients compared to their counterparts (Adjusted hazard ratio (AHR) = 1.43, 95% confidence interval (CI): 1.05–1.95) (Table 3). Furthermore, unplanned readmission (UR) incidence is higher by 62% in women with no English-speaking background (AHR = 1.62, 95% CI: 1.07–2.47). Length of hospital stay with more than 3 days was associated with increased UR (AHR = 1.41, 95% CI: 1.04–1.90). The patient’s education level and ownership of private insurance were not significantly related to the risk of UR in the multivariate analysis. Living with one or two chronic disease(s) was associated with an increased risk of unplanned readmission. The UR is higher by 68% among women living with one or two chronic diseases compared to women who have no chronic disease (AHR = 1.68, 95% CI: 1.19–2.36). The ratio of hazards within the covariates category is constant in time which did not violate the proportional hazard. The sensitivity analysis was conducted by including the length of time between the women’s most recent survey (which will vary according to the women) and the date of index admission (Table S1). The length of time was not significant and did not make a difference on other covariate’s estimate.

## 4. Discussion

This study identified the rate of unplanned readmission and associated factors among older Australian women who were followed for over fifteen years in the Australian Longitudinal Study on Women’s Health (ALSWH) [22]. Overall, 11.14% of women had an unplanned readmission within 28 days of hospital discharge. This figure is higher than the findings from the previous study in Australia [5,9]. For instance, a retrospective study from a major Australian health service showed that the unplanned readmission rate was 10.1%% in older people aged 65 years and over [5]. However, our finding is lower than 14.4% and 14.0% thirty-day readmission rate among community-dwelling seniors visiting hospital emergency departments, at the University of Pennsylvania, USA [3] and the province of Quebec, Canada, respectively [2].

Unplanned readmission within a few weeks of hospital discharge is a major burden to the healthcare system and a leading topic of healthcare policy and practice [32]. In Australia, unplanned hospital readmission within 28 days of hospital discharge is deemed as an indicator of quality and safety of healthcare. Promoting strategies targeting the reduction of readmission is crucial to alleviate the risk of unplanned readmission. For instance, a quasi-experimental retrospective design study in Australia showed that a phone call following hospital discharge reduced 28 days readmissions by 29% [33]. Another study showed that transitional care can effectively reduce readmissions among older Australians [34]. Finlayson et al. showed 28 days unplanned readmissions were reduced by 11% for older people who received combined exercise and nurse follow-up intervention [34]. With comprehensive discharge planning and post-discharge follow-up strategies, it would be possible to reduce unplanned readmission in older populations. Other successful strategies include improving inpatient care, advanced discharge planning, better access to outpatient care, and improved community support [35]. Applied interventions in previous research include improving discharge plan, patient education, post-discharge telephone follow-up, home visits or a combination [36,37]. A systematic review of interventions to reduce hospital readmission in the elderly concluded that interventions that included home care components seem to be more likely to reduce readmissions in the older population [38].

This study showed unpartnered women were more likely to experience unplanned readmission within 28 days of discharge. Several studies showed living alone and/or being single has been a risk factor of adverse outcomes and/or early readmission in the older population following an episode of hospital care [34,39,40]. Likewise, a Swedish study that included older Swedish participants concluded living alone was associated significantly to a high risk of unplanned hospitalisation in the elderly [40]. This could be due to lack of care at home, social isolation, inability to discuss health needs with others or lack of support in activities of daily living [41]. Body mass index was not a significant predictor of unplanned readmission in our study, or in another study [42]. Smoking was not significantly related to readmission once other factors were included in the models, but smoking may be associated with multiple morbidities.

Importantly, women residing in regional and remote areas had a high risk of readmission compared to women living in metropolitan areas in the univariate analysis only, although it did not remain in the final model. The previous study highlighted insufficient health access and poor health outcomes in people living in rural areas [43]. Future studies may need to evaluate drivers of unplanned readmission and health service use of people living in remote areas. This study revealed that not speaking English as a primary language at home was significantly associated with higher unplanned readmission rates. Another study from the USA showed that patients with limited English proficiency were 24% more likely to experience readmission in the emergency department within 72 h of discharge compared to their counterparts [44]. Improving the discharge plan of patients who do not speak English as their primary language at home or with limited English proficiency may be important to reduce avoidable unplanned readmission following discharge from hospital [44].

A longer stay in hospital among older people was also associated with a high risk of readmission in this study. Women who stayed longer during index admission may have more complex health issues compared to those with shorter hospital stays. This study’s findings are consistent with previous literature that showed older people with a chronic disease are more likely to experience the unplanned readmission within a few weeks of hospital discharge [5,11,45]. Living with one or two chronic diseases is one of the significant risk factors for unplanned readmission. Consistently, a previous study showed French older people living with morbidity were at higher risk of readmission [46].

One of the limitations of this study is the lack of factors at the level of the health service provider. Although unplanned readmission is an indicator of the quality and safety of healthcare, this study did not include the potential predictors at the healthcare provider level. For instance, another study showed that patients in health service sites that manage surgical and medical patients with a higher level of complexity have had higher unplanned readmission risk in 28 days [5]. The other limitation is that only older women were included in this study, which needs attention in interpreting this study result. Baseline characteristics have been used from the latest self-reported survey before the index admission date. By considering this limitation, additional sensitivity analysis was conducted by including the length of time between the women’s self-reported survey return date and the index hospital admission date. This did not make any difference on the estimate of predictors of unplanned readmission. Given these potential limitations, this study has numerous strengths. One of the main strengths of this study is that we used a longitudinal study (ALSWH), which assessed prospectively older women’s physical and mental health and related health service use. This study is the first study, which examined predictors of unplanned readmission in older women using a prospective study, conducted over fifteen years.

## 5. Conclusions

Although there is a lack of clarity in defining unplanned readmission across the literature, this study reported a considerable risk of unplanned readmission within 28 days of discharge among older women. At least one in ten women had unplanned readmission at some time between ages 75–95 years. Women who were not partnered, not English-speaking background, with a longer stay in hospital and living with a chronic disease in older age were associated with increased risk of unplanned readmission. Interventions aiming at those who are not partnered, are not principally English speaking, have longer hospital stays and are living with a chronic disease are essential to alleviate unplanned readmission risk.

## Figures and Tables

**Table 1:** Baseline characteristics and readmission incidence among older women aged 75 years and over from 1921 to 1926 birth cohort of ALSWH.

Baseline Characteristics	No Readmission (*n* = 1693) *n* (%)	Planned Readmission (*n* = 134) *n* (%)	Unplanned Readmission (*n* = 229) *n* (%)	*p*-Value
*Predisposing factors*				
Age at index admission, mean ± SD	82.11 ± 3.95	81.65.00 ± 3.75	82.00 ± 4.18	0.546
Marital status				
Partnered	687 (40.67)	46 (34.33)	66 (28.82)	0.001
Not partnered	1002 (59.33)	88 (65.67)	163 (71.18)	
Missing	4			
English speaking				
No	1452 (90.02)	110 (84.09)	185 (84.09)	0.015
Yes	161 (9.98)	18 (14.06)	35 (15.91)	
Missing	80	6	229	
*Enabling factors*				
Area				
Metropolitan	709 (41.88)	61 (45.52)	77 (33.62)	0.130
Inner regional	696 (41.11)	53 (39.55)	105 (45.85)	
Outer regional/remote/very remote	288 (17.01)	20 (14.93)	47 (20.52)	
Education				
Less than High school	1248 (77.52)	95 (74.80)	169 (77.17)	0.676
School certificate	222 (13.79)	16 (12.60)	29 (13.24)	
Higher/above school certificate	140 (8.7)	16 (12.60)	21 (9.59)	
Missing	83	7	10	
Private insurance				
No	1046 (62.37)	75 (56.39)	154 (67.84)	0.087
Yes	631 (37.63)	58 (43.61)	73 (32.16)	
Missing	16	1	2	
*Need factors*				
Smoking				
Non-smoker	1035 (65.46)	76 (61.29)	140 (64.81)	0.038
Ex-smoker	444 (28.08)	31 (25.00)	57 (26.39)	
Current smoker	102 (6.45)	17 (13.71)	19 (8.80)	
Missing	112	10	13	
LOS index				
≤3 days	764 (45.13)	59 (44.03)	85 (37.12)	0.073
>3 days	929 (54.87)	75 (55.97)	144 (62.88)	
BMI				
Underweight	71 (4.41)	2 (1.56)	9 (4.04)	0.137
Normal weight	806 (50.06)	68 (53.13)	103 (46.19)	
Overweight	495 (30.75)	48 (37.50)	77 (34.53)	
Obese	238 (14.78)	10 (7.81)	34 (15.25)	
Missing	83	6	6	
Perceived general health				
Good/excellent	1075 (64.29)	86 (64.66)	147 (64.19)	0.995
Poor/not good	597 (35.71)	47 (35.34)	82 (35.81)	
Missing	21	1		
GP or family doctor visit				
≤4	554 (36.76)	42 (34.15)	81 (41.97)	0.287
>4	953 (63.24)	81 (65.85)	112 (58.03)	
Missing	186	11	36	
Chronic disease				
No	526 (34.90)	44 (35.77)	51 (25.89)	0.026
1–2	636 (42.20)	56 (45.53)	106 (53.81)	
>2	345 (22.89)	23 (18.70)	40 (20.30)	
Missing	186	11	32	

Pearson’s chi-square was performed to compare patients’ characteristics with readmission incidence and Kruskal-Wallis test was used for a covariate age. SD—standard deviation; LOS—length of hospital stay; BMI—body mass index; GP—general practitioner.

**Table 2:** Top five-index admission diagnosis with the corresponding top three unplanned readmission diagnosis among older women aged 75 years and over from 1921 to 1926 birth cohort of ALSWH.

Top Five Index Admission	Unplanned Readmission	Definition
I30–I52 (13.98%)	I30–I52 (31.25%)	Other forms of heart disease
	R50–R69 (9.38%)	General symptoms and signs
	I70–I79 (6.25%)	Arterial embolism and thrombosis
	Other (53.13%)	
I20–I25 (7.42%)	I20–I25 (35.29%)	Ischaemic heart disease
	I30–I52 (23.53%%)	Other forms of heart disease
	J09–J18 (11.76%)	Influenza and pneumonia
	Other (29.42%)	
R00–R09 (4.80%)	R00–R09 (18.18%)	Symptoms and signs involving the circulatory and respiratory systems
	Other (81.82%)	
K80–K87 (4.37%)	K80–K87 (33.33%)	Disorder of gallbladder, biliary tract and pancreas
	Other (76.67%)	
K55–K64 (4.37%)	K55–K64 (33.33%)	Other diseases of intestines
	Other (76.67%)	

**Table 3:** Univariate and multivariate analysis showing the association between patient characteristics and unplanned readmission episodes within 28 days of hospital discharge among older women aged 75 years and over from 1921 to 1926 birth cohort of ALSWH (2001–2016).

Characteristics	Unplanned Readmission *n* (%) (*n* = 229)	Univariate Analysis HR (95% CI)	*p*-Value	Multivariate Analysis AHR (95% CI)
*Predisposing factors*				
Age	82.30 ± 4.18	1.01 (0.98, 1.05)	0.584	1.02 (0.98, 1.06)
Marital status (Ref: Partnered)	66 (28.82)	1	<0.01	1
Not Partnered	163 (71.18)	1.61 (1.21, 2.14) **		1.43 (1.05, 1.95) *
English speaking (Ref: Yes)	185 (84.09)	1	0.012	1
No	35 (15.91)	1.59 (1.11, 2.28) *		1.62 (1.07, 2.47) *
*Enabling factor*				
Area (Ref: Metropolitan)	77 (33.62)	1	0.043	1
Inner regional	105 (45.85)	1.37 (1.02, 1.84) *		1.28 (0.92, 1.78)
Outer regional/remote/very remote	47 (20.52)	1.50 (1.04, 2.15) *		1.47 (0.97, 2.22)
Education (Ref: Higher/above school certificate)	169 (77.17)	1	0.947	
School certificate	238 (13.24)	0.97 (0.65, 1.44)		
Less than high school	21 (9.59)	1.07 (0.68, 1.68)		
Private insurance (Ref: Yes)	154 (67.84)	1	0.085	1
No	73 (32.16)	0.78 (0.59, 1.04)		0.79 (0.58, 1.08)
*Need factors*				
Smoking (Ref: Non-smoker)	140 (64.81)	1	0.609	
Ex-smoker	57 (26.39)	0.96 (0.71, 1.31)		
Current smoker	19 (8.80)	1.25 (0.77, 2.01)		
LOS in index (Ref: ≤3)	85 (37.12)	1	0.026	1
Greater than 3	144 (62.88)	1.36 (1.04, 1.77) *		1.41(1.04, 1.90) *
BMI (Ref: Normal weight)	10 (4.48)	1	0.709	
Underweight	100 (44.84)	0.95 (0.48, 1.88)		
Overweight	78 (34.98)	1.13 (0.56, 2.25)		
Obese	35 (15.70)	1.09 (0.52, 2.27)		
Perceived general health (Ref: Good/excellent)	127 (64.47)	1	0.971	
Poor/not good	70 (35.53)	1.01 (0.77, 1.31)		
GP/family doctor visit (Ref: ≤4)	81 (41.97)	1	0.123	1
>4	112 (58.03)	0.81 (0.63, 1.06)		0.82 (0.61, 1.11)
Chronic disease (Ref: No)	51 (25.89)	1	0.007	1
1–2	106 (53.81)	1.66 (1.19, 2.32) *		1.68 (1.19, 2.36) *
>2	40 (20.30)	1.21 (0.79, 1.83)		1.18 (0.77, 1.83)

* is significant covariate at 5% significance level; ** is significant covariate at 1% significance level. HR—hazard ratio; AHR—adjusted hazard ratio; CI—confidence interval; Ref—reference; LOS—length of hospital stay; BMI—body mass index; GP—general practitioner.

## Supplementary Materials

The following are available online at https://www.mdpi.com/1660-4601/17/9/3136/s1, Table S1: Sensitivity analysis showing the patient’s characteristics and length of time (between survey return date and index admission) association with unplanned readmission episode within 28 days of hospital discharge among older women aged 75 years and over from 1921 to 1926 birth cohort of ALSWH (2001–2016).
