# Supplementary material for: Unplanned Readmission within 28 Days of Hospital Discharge in a Longitudinal Population-Based Cohort of Older Australian Women
Source: Int J Environ Res Public Health. 2020 Apr 30;17(9):3136. doi: 10.3390/ijerph17093136 (PMC7246843; doi:10.3390/ijerph17093136)
Supplement: Supplementary file 1 [file ijerph-17-03136-s001.pdf]

Table S1. Sensitivity analysis showing the patient's characteristics and length of time (between survey return date and index admission) association with unplanned readmission episode within 28 days of hospital discharge among older women aged 75 years and over from 1921 to 1926 birth cohort of ALSWH (2001–2016).

Characteristics	Univariate Analysis HR (95% CI)	*p*-Value	Multivariate Analysis AHR (95% CI)	Multivariate Analysis AHR (95% CI) After Time Adjustment
Predisposing factors				
Woman's survey returned (Ref= before 3 years before admission)	1			1
>3 years before admission	1.12 (0.85, 1.49)	0.117		0.82 (0.55, 1.21)
Age	1.01 (0.98, 1.05)	0.584	1.02 (0.98, 1.06)	1.03 (0.99, 1.07)
Marital status (Ref: Partnered)	1	<0.01	1	1
Not Partnered	1.61 (1.21, 2.14) **		1.43 (1.05, 1.95) *	1.42 (1.04, 1.94) *
English speaking (Ref: Yes)	1	0.012	1	1
No	1.59 (1.11, 2.28) *		1.62 (1.07, 2.47) *	1.65 (1.08, 2.51) *
Enabling factor				
Area (Ref: Metropolitan)	1	0.043	1	1
Inner regional	1.37 (1.02, 1.84) *		1.28 (0.92, 1.78)	1.27 (0.91, 1.77)
Outer regional/remote/very remote	1.50 (1.04, 2.15) *		1.47 (0.97, 2.22)	1.46 (0.97, 2.21)
Education (Ref: Higher/above school certificate)	1			
School certificate	0.97 (0.65, 1.44)	0.947		
Less than high school	1.07 (0.68, 1.68)			
Private insurance (Ref: Yes)	1	0.085	1	1
No	0.78 (0.59, 1.04)		0.79 (0.58, 1.08)	0.79 (0.57, 1.08)
Need factors				
Smoking (Ref: Non-smoker)	1			
Ex-smoker	0.96 (0.71, 1.31)	0.609		
Current smoker	1.25 (0.77, 2.01)			
LOS in index (Ref: ≤3)	1	0.026	1	1
Greater than 3	1.36 (1.04, 1.77) *		1.41 (1.04, 1.90) *	1.41 (1.04, 1.90) *
BMI (Ref: Normal weight)	1			
Underweight	0.95 (0.48, 1.88)	0.709		
Overweight	1.13 (0.56, 2.25)			
Obese	1.09 (0.52, 2.27)			

Perceived general health (Ref: Good/excellent)	1	0.971		
Poor/not good	1.01 (0.77, 1.31)			
GP/family doctor visit (Ref: ≤4)	1	0.123	1	1
>4	0.81 (0.63, 1.06)		0.82 (0.61, 1.11)	0.82 (0.61, 1.11)
Chronic disease (Ref: No)	1		1	1
1–2	1.66 (1.19, 2.32) *	0.007	1.68 (1.19, 2.36) *	1.66 (1.18, 2.34) *
>2	1.21 (0.79, 1.83)		1.18 (0.77, 1.83)	1.19 (0.77, 1.84)

* is significant covariate at 5% significance level; ** is significant covariate at 1% significance level. HR—hazard ratio; AHR—adjusted hazard ratio; CI—confidence interval; Ref—reference; LOS—length of hospital stay; BMI—body mass index; GP—general practitioner.
